# Surrogate endpoints in advanced sarcoma trials: a meta-analysis

**DOI:** 10.18632/oncotarget.26166

**Published:** 2018-10-02

**Authors:** Marion Savina, Saskia Litière, Antoine Italiano, Tomasz Burzykowski, Franck Bonnetain, Sophie Gourgou, Virginie Rondeau, Jean-Yves Blay, Sophie Cousin, Florence Duffaud, Hans Gelderblom, Alessandro Gronchi, Ian Judson, Axel Le Cesne, Paul Lorigan, Joan Maurel, Winette van der Graaf, Jaap Verweij, Simone Mathoulin-Pélissier, Carine Bellera

**Affiliations:** ^1^ Clinical and Epidemiological Research Unit, Institut Bergonié, Comprehensive Cancer Center, Bordeaux cedex 33076, France; ^2^ INSERM CIC-EC 14.01 (Clinical Epidemiology), Bordeaux 33000, France; ^3^ INSERM, ISPED, Centre INSERM U1219 Bordeaux Population Health Center, Epicene Team, Bordeaux 33000, France; ^4^ University of Bordeaux, ISPED, Centre INSER M U1219 Bordeaux Population Health, Epicene Team, Bordeaux 33000, France; ^5^ European Organisation for Research and Treatment of Cancer (EORTC), Brussels 1200, Belgium; ^6^ Medical Oncology Unit, Institut Bergonié, Comprehensive Cancer Center, Bordeaux cedex 33076, France; ^7^ Interuniversity Institute for Biostatistics and Statistical Bioinformatics (I-BioStat), Hasselt University, Hasselt 3500, Belgium; ^8^ Methodology and Quality of life in Oncology Unit, Besançon EA3181, France; ^9^ Biometrics Unit, Institut du Cancer de Montpellier, Univ. Montpellier, Montpellier 34298, France; ^10^ INSERM, ISPED, Centre INSERM U1219 Bordeaux Population Health Center, Biostatistic Team, Bordeaux 33000, France; ^11^ Centre Léon Bérard, Comprehensive Cancer Center, Lyon 69008, France; ^12^ University Claude Bernard Lyon I, Lyon 69000, France; ^13^ Medical Oncology Unit, University Hospital La Timone and University of Aix-Marseille, Marseille 13005, France; ^14^ Department of Medical Oncology, Leiden University Medical Center, Leiden 2300RC, The Netherlands; ^15^ Sarcoma Service, Department of Surgery, Fondazione IRCCS Istituto Nazionale dei Tumori, Milano, Italy; ^16^ Institute of Cancer Research, Sutton, Surrey, United Kingdom; ^17^ Medicine Department, Institut Gustave Roussy, Comprehensive Cancer Center, Villejuif 94800, France; ^18^ University of Manchester and Christie NHS Foundation Trust, Manchester M20 4BX, UK; ^19^ Department of Medical Oncology, Hospital Clinic, CIBERehd, Translational Genomics and Targeted Therapeutics in Solid Tumors (IDIBAPS), Barcelona 08036, Spain; ^20^ The Institute of Cancer Research, Sutton, London SM2 5NG, United Kingdom; ^21^ Radboud University Medical Centre, Department of Medical Oncology, GA Nijmegen 6525, The Netherlands; ^22^ Department of Medical Oncology, Erasmus University Medical Center, CE Rotterdam 3015, The Netherlands; ^23^ Royal Marsden NHS Foundation Trust, Chelsea, London, United Kingdom

**Keywords:** sarcoma, surrogate endpoints, meta-analysis, randomized trial, survival

## Abstract

**Background:**

Alternative endpoints to overall survival (OS) are frequently used to assess treatment efficacy in randomized controlled trials (RCT). Their properties in terms of surrogate outcomes for OS need to be assessed. We evaluated the surrogate properties of progression-free survival (PFS), time-to-progression (TTP) and time-to-treatment failure (TTF) in advanced soft tissue sarcomas (STS).

**Results:**

A total of 21 trials originally met the selection criteria and 14 RCTs (*N* = 2846) were included in the analysis. Individual-level associations were moderate (highest for 12-month PFS: Spearman’s rho = 0.66; 95% CI [0.63; 0.68]). Trial-level associations were ranked as low for the three endpoints as per the IQWiG criterion.

**Materials and Methods:**

We performed a meta-analysis using individual-patient data (IPD). Phase II/III RCTs evaluating therapies for adults with advanced STS were eligible. We estimated the individual- and the trial-level associations between then candidate surrogates and OS. Statistical methods included weighted linear regression and the two-stage model introduced by Buyse and Burzykowski. The strength of the trial-level association was ranked according to the German Institute for Quality and Efficiency in Health Care (IQWiG) guidelines.

**Conclusions:**

Our results do not support strong surrogate properties of PFS, TTP and TTF for OS in advanced STS.

## INTRODUCTION

The choice of the primary endpoint is a key step when designing a randomized controlled trial (RCT). In oncology, the most commonly used endpoint to assess the efficacy of a new treatment in RCTs is overall survival (OS) which is easily measurable, objectively defined as the time from randomization to death and validated by health regulatory authorities [1]. Alternative time-to-event endpoints are commonly used in practice in phase II trials and increasingly being used instead of OS in phase III trials [2]. These composite endpoints include biological and clinical events, such as disease progression or treatment toxicity. Their development is driven by the need to reduce the number of patients, the trial duration, the delay to reach conclusions and ultimately the cost of the trials. However, it is essential to rigorously assess their surrogate properties for OS, and as such whether or not they can be used as primary endpoints for assessing the benefit of new therapies. This approach does not preclude their intrinsic value as parameters of patient benefit of a treatment.

As of today, the meta-analytic surrogacy evaluation scheme proposed by Buyse and Burzykowski *et al.* [3, 4] is considered as the most statistically rigorous method for the validation of surrogate endpoints [5, 6]. This approach requires individual-patient data (IPD) from multiple RCTs with similar design and treatment to address surrogacy from a multi-level framework. At the patient level, the surrogate endpoint should be correlated and predictive of the final endpoint regardless of the treatment (individual-level association). At the trial level, the treatment effect on the surrogate endpoint should be correlated and predictive of the treatment effect on the final endpoint (trial-level association).

Soft-tissue sarcomas (STS) are a heterogeneous group of diseases that account for 1% of all malignancies in adults [7]. Despite adequate locoregional treatment, up to 40% of patients with STS develop metastatic disease [7]. When metastases are detected, the standard of care is palliative chemotherapy. Due to their rarity, conducting large RCTs to evaluate the benefit of new treatment for metastatic STS is complex. The identification of valid surrogate endpoints for OS that would reduce the number of included patients would be of a great advantage for clinical research. To our knowledge, only one meta-analysis evaluating response rate and PFS as surrogates for OS in metastatic STS was conducted [8]. The study was however limited to the analysis of aggregated data.

We performed a meta-analysis of RCTs using IPD to assess the surrogate properties for OS of three commonly used endpoints in advanced STS: progression-free survival (PFS), time-to-progression (TTP) and time-to-treatment failure (TTF). This manuscript follows the international recommendations of the PRISMA guidelines for reporting meta-analyses [9].

## RESULTS

### Data

**Figure 1:**
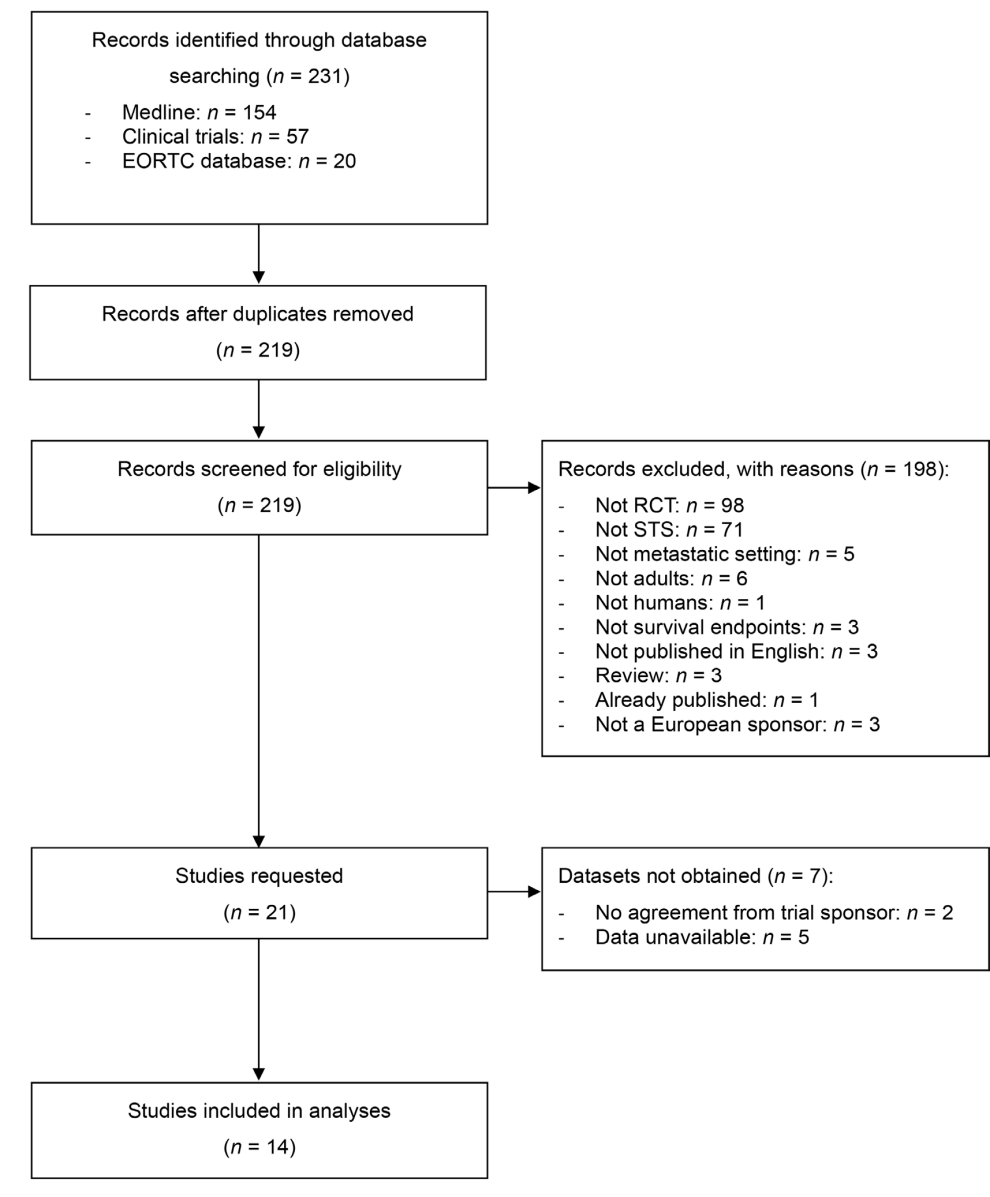
Flow of information through the different phases of the study selection, as per PRISMA guidelines [9]^*^ ^*^EORTC = European Organisation for Research and Treatment of Cancer; RCT = randomized controlled trial; STS = soft-tissue sarcoma.

After screening 231 abstracts, we identified 21 eligible trials and obtained the trial sponsor’s agreement for 19 RCTs (Figure 1). IPD were available for 14 RCTs [10–23]. Trials characteristics are presented in Table 1. Three trials had two experimental arms evaluating different administration schedules for the same drug [10, 15, 23]. We combined the two experimental arms into one for these studies. One trial was designed as two parallel randomized trials [14], it was included in the meta-analysis as two distinct trials so that we considered a total of 15 trials. Aside from one trial [18], RCTs evaluated chemotherapy-based regimens. Most trials compared an experimental chemotherapy to a doxorubicin or ifosfamide-based chemotherapy regimen as first-line treatment (Table 1). One trial focused on leiomyosarcomas [20] and one trial excluded liposarcomas [23], all 13 other trials presented similar histological subtypes. Out of the 14 trials included, 11 relied on radiological central review at study entry and two trials used histology review (local or in a specialized center).

**Table 1:** Key characteristics of the included trials*

Study	Ref	Phase	Inclusion period	*N*	Treatment line	Control arm	Experimental arm	Median follow-up	
								All patients	Patients alive
62901	[10]	II	≥1991	334	1st line	Doxorubicin	Epirubicin	50.2 months	13.3 months
62941	[11]	II	≥1995	86	1st and 2nd line	Doxorubicin	Docetaxel	35.9 months	10.9 months
62903	[12]	III	1992–1995	315	1st line	Doxorubicin + Ifosfamide	Doxorubicin + Ifosfamide + GM-GSF	91.4 months	40.1 months
62962	[13]	II	≥1997	95	1st line	Doxorubicin	Doxorubicin pegylated liposomal	35.2 months	14.5 months
62912	[14]	II	1992–1994	78	2nd-line	Ifosfamide5 g/m^2^/1 day	Ifosfamide3 g/m^2^/3 days	30.6 months	16.2 months
			1994–1996	103	1st line	Ifosfamide5 g/m^2^/1 day	Ifosfamide3 g/m^2^/3 days	35.5 months	6.6 months
62971	[15]	III	1998–2001	326	1st line	Doxorubicin	Ifosfamide	51.7 months	43.1 months
GEIS9	[16]	II	2003–2007	132	1st line	Doxorubicin	Intensified Doxorubicin + Ifosfamide	22.5 months	15.4 months
Palsar 1	[17]	III	1994–1997	145	1st line	MAID	Intensified MAID	93.0 months	89.7 months
62072	[18]	III	2008–2010	369	2nd to 5th line	Placebo	Pazopanib	14.6 months	12.2 months
Palsar 2	[19]	III	2000–2008	87	1st line	MAID	MAID + MICE	22.3 months	21.4 months
Taxogem †	[20]	II	2006–2008	70	2nd line	Gemcitabine	Gemcitabine + Docetaxel + Lenograstime	32.5 months	24.9 months
62012	[21]	III	2003–2010	455	1st line	Doxorubicin	Intensified Doxorubicin + Ifosfamide	56.4 months	30.7 months
62061	[22]	II	2006–2008	118	1st line	Doxorubicin	Brostallicin	21.3 months	19.3 months
62091	[23]	IIb/III	≥2011	133	1st line	Doxorubicin	Trabectedin	9.4 months	8.6 months

^*^GM-GSF: Recombinant human granulocyte-macrophage colony-stimulating factor; MAID = Doxorubicin, Ifosfamide and Dacarbazine; MICE: Mesna, Ifosfamide, Carboplatin and Etoposide; Ref = reference.

^†^Only patients with leiomyosarcoma included.

**Figure 2:**
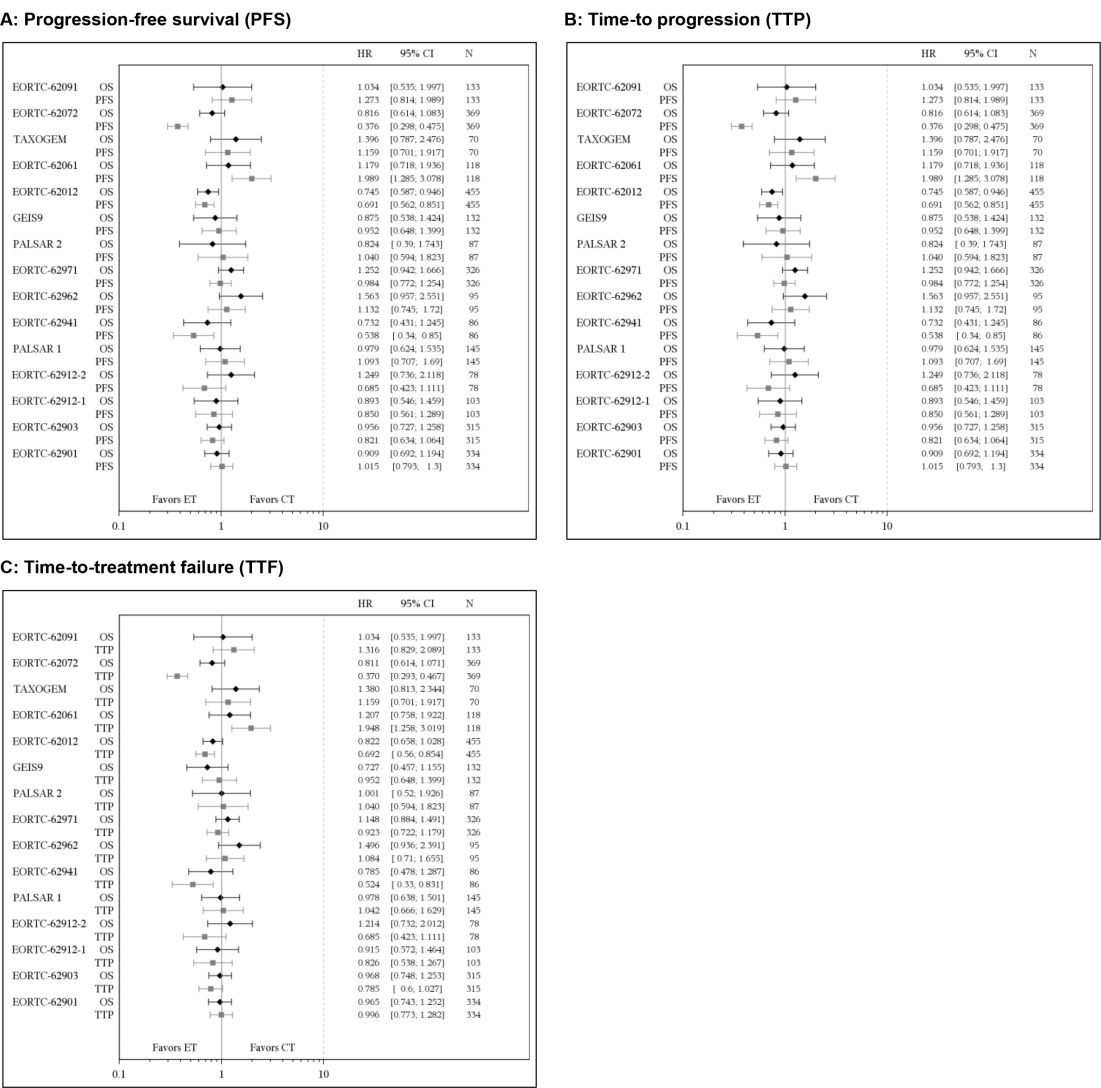
Forest plots ^*^Treatment effects on 12-month progression-free survival (**A**), time-to progression (**B**) and time-to-treatment failure (**C**) and on 18-month overall survival (OS) estimated by hazard ratios (HR) using separate Cox models. The first row for each trial shows the result for OS, and the second row shows the result for the candidate surrogate. The diamonds and squares represent the point estimates for OS and the candidate surrogate, respectively. The horizontal error bars show the 95% confidence interval (CI) of each hazard ratio (15 trials, 2846 patients). CT = control treatment; ET = experimental treatment.

IPD from the 2846 patients included in the trials were analyzed. Median follow-up duration ranged from 9 to 93 months (median: 36 months). Figure 2 displays forest plots for the treatment effects estimated by hazard ratios (HR) on two-year OS, and one-year PFS, TTP and TTF for each trial. Among the 2165 patients who died during the total follow-up, 1704 (79%) died during the first 18 months. For each of the three candidate surrogate endpoints, the number of events observed at 6 and 12 months is provided in Supplementary Table 1.

### Correlation between the candidate surrogate endpoints and OS (individual-level surrogacy)

We relied on a one-parameter Clayton copula model, considered the best fitting model compared to Plackett or Hougaard copula. Considering a six-month follow-up for the surrogate endpoints, the individual-level correlations with 18-month OS were moderate, with PFS showing the highest correlation (0.62; 95% CI [0.59; 0.65]) (Table 2). Correlations obtained when using a one-year follow-up for the surrogate endpoints were also moderate, even though slightly higher (PFS: 0.66; 95% CI [0.63; 0.68]).

**Table 2:** Individual- and trial-level associations between 6-month and 12-month progression-free survival, time-to-progression, time-to-treatment failure and 18-month overall survival*

		Individual-level association	Trial-level association	
Folow-up	Endpoint	ρ_Spearman_^†^ [95% CI]	R^2^_WLR_^‡^ [95% CI]	R^2^_2SM_^φ^ [95% CI]
All trials (*N*_trial_ = 15; *N*_patient_ = 2846)				
6 months	PFS	0.62 [0.59; 0.65]	0.33 [0.00; 0.60]	0.04 [0.00; 0.43]
	TTP	0.59 [0.56; 0.63]	0.32 [0.00; 0.58]	0.07 [0.00; 0.60]
	TTF	0.60 [0.57; 0.63]	0.32 [0.00; 0.58]	0.06 [0.00; 0.57]
12 months	PFS	0.66 [0.63; 0.68]	0.33 [0.00; 0.60]	0.00 [0.00; 0.05]
	TTP	0.63 [0.60; 0.66]	0.30 [0.00; 0.57]	0.00 [0.00; 0.02]
	TTF	0.64 [0.61; 0.67]	0.31 [0.00; 0.58]	0.00 [0.00; 0.01]
Doxorubicin- or ifosfamide-based treatment, first-line setting (*N*_trial_ = 11; *N*_patient_ = 2243)				
6 months	PFS	0.63 [0.60; 0.67]	0.30 [0.00; 0.60]	0.00 [0.00; 0.08]
	TTP	0.60 [0.56; 0.64]	0.26 [0.00; 0.58]	0.00 [0.00; 0.11]
	TTF	0.61 [0.57; 0.65]	0.27 [0.00; 0.58]	0.00 [0.00; 0.06]
12 months	PFS	0.67 [0.64; 0.70]	0.39 [0.00; 0.66]	0.08 [0.00; 0.86]
	TTP	0.64 [0.61; 0.68]	0.31 [0.00; 0.61]	0.12 [0.00; 1.00]
	TTF	0.65 [0.62; 0.68]	0.32 [0.00; 0.62]	0.10 [0.00; 1.00]
Leiomyosarcomas (*N*_trial_ = 14; *N*_patient_ = 1025)				
6 months	PFS	0.57 [0.51; 0.62]	0.59 [0.15; 0.76]	0.91 [0.00; 1.00]
	TTP	0.55 [0.49; 0.60]	0.58 [0.13; 0.75]	0.97 [0.00; 1.00]
	TTF	0.53 [0.48; 0.58]	0.59 [0.14; 0.76]	0.91 [0.00; 1.00]
12 months	PFS	0.59 [0.54; 0.64]	0.59 [0.16; 0.75]	0.91 [0.00; 1.00]
	TTP	0.52 [0.47; 0.58]	0.58 [0.15; 0.75]	0.97 [0.00; 1.00]
	TTF	0.53 [0.48; 0.58]	0.58 [0.15; 0.75]	0.91 [0.00; 1.00]

^*^CI = confidence interval; PFS = progression-free survival; TTF = time-to-treatment failure; TTP = time-to-progression.

^†^ρSpearman represents the Spearman rank correlation coefficient between the candidate surrogates and overall survival.

^‡^R^2^WLR represents the coefficient of determination between treatment effect on the candidate surrogates and overall survival based on weighted linear regression models.

^φ^R^2^2SM represents the coefficient of determination between treatment effect on the candidate surrogates and overall survival based on the two-stage model [4].

### Correlation between treatment effects on the candidate surrogate endpoints and treatment effect on OS (trial-level surrogacy)

**Figure 3:**
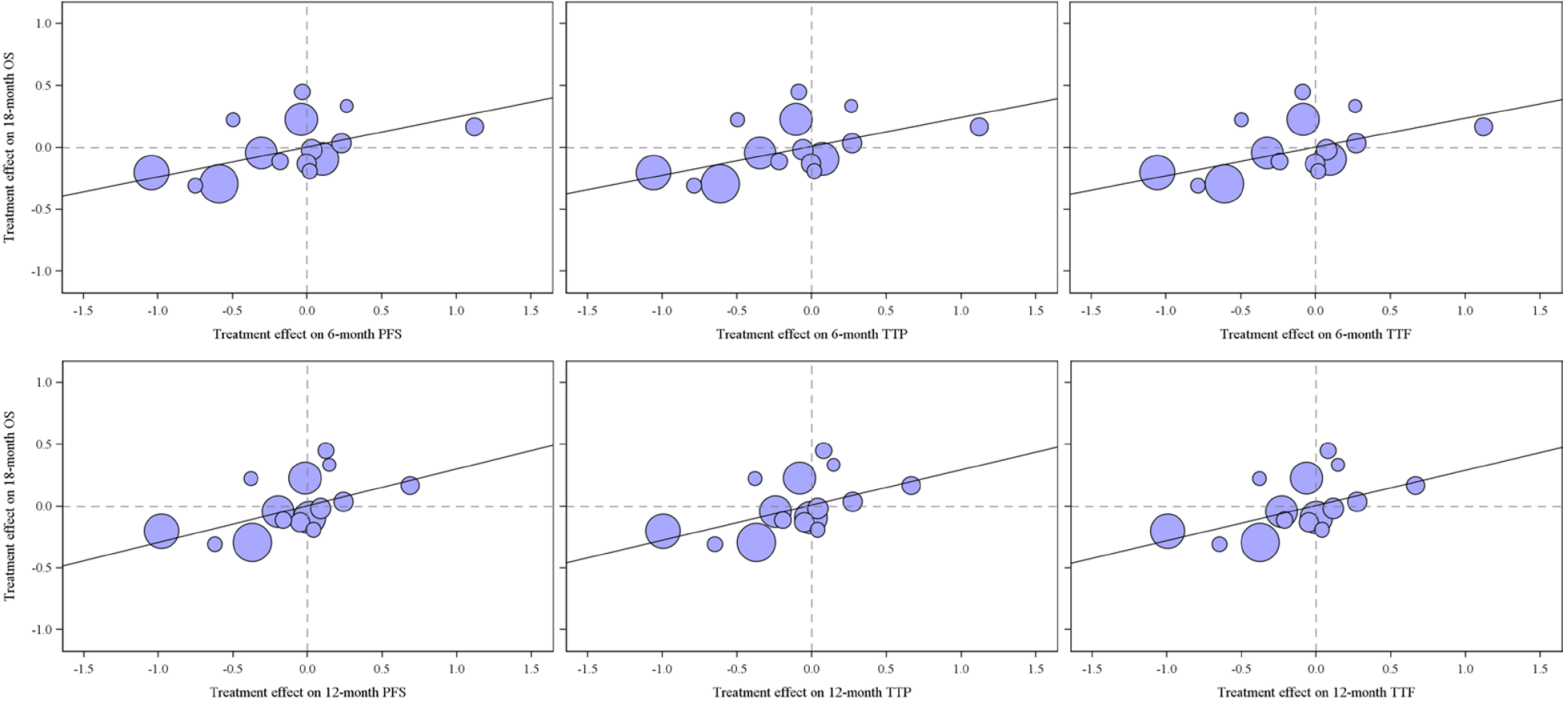
Trial-level association between treatment effects on the candidate surrogates and overall survival^*^ ^*^Treatment effects estimated by the logarithm of hazard ratios (log[HR]) using the weighted linear regression approach. Each circle represents a trial, and the surface area of the circle is proportional to the size of the corresponding trial. PFS = progression-free survival; TTF = time-to-treatment failure; TTP = time-to-progression; OS = overall survival.

A total of 15 pairs of log(HR) were compared for each endpoint. When considering a six-month follow-up for the surrogates, the trial-level associations R^2^_WLR_ and R^2^_2SM_, were low (R^2^_WLR_ ≤ 0.60; R^2^_2SM_ ≤ 0.60) (Table 2). When considering a one-year follow-up, the association measures remained low (R^2^_WLR_ ≤ 0.60; R^2^_2SM_ ≤ 0.05). Regression curves calculated based on the WLR models are shown in Figure 3. As per IQWiG guidelines, all trial-level associations estimated were ranked as medium.

### Subgroup analyses

The first subgroup analysis focused on trials comparing systemic therapy to doxorubicin- or ifosfamide-based chemotherapies in the first-line setting (*N*_trial_ = 11; *N*_patient_ = 2243). When considering a six-month follow-up, the three endpoints were moderately associated with 18-month OS at the patient level (0.56 ≤ ρ_Spearman_ ≤ 0.67). At the trial level, the association between the candidate surrogates and 18-month OS was low (R^2^_WLR_ ≤ 0.60; R^2^_2SM_ ≤ 0.11) (Table 2). When considering a 12-month follow-up, the individual-level and trial-level associations increased. Confidence intervals for trial level associations were large, irrespective of the statistical method used. The number of events observed in the subgroup of trials at 6 and 12 months is provided in Supplementary Table 2. For the second subgroup analysis focusing on leiomyosarcomas, the treatment effects on OS and on the candidate surrogates could not be estimated for one trial due to a lack of events, it was then excluded from the subgroup analysis. Individual-level correlations slightly decreased compared to the primary analysis. At the trial level however, the correlations significantly increased (Table 2), although again, confidence interval were large.

## DISCUSSION

We pooled IPD data from 2846 patients included in 14 RCTs to evaluate the surrogate properties of PFS, TTP and TTF for OS in advanced STS. At the individual-level, associations between the three endpoints and OS were moderate, with the highest correlation observed for PFS. At the trial level, associations between the treatment effects on three endpoints and treatment effect on OS were low with wide confidence intervals. The strength of the trial-level association was quantified as medium as per the IQWiG criteria, indicating that the validity of the endpoints as surrogates for OS remains unclear.

Several statistical methods are available to assess surrogacy. We relied on the two-stage approach developed by Buyse and Burzykowski based on IPD [4], considered the most rigorous statistical approach for surrogacy assessment [5, 6]. Similarly, several criteria have been proposed to assess the validity of surrogate endpoints [24–26]. Although they present differences, they all require a lower limit of the 95% CI for the trial-level correlation coefficient at least higher than 0.6 to definitely validate a surrogate endpoint. As such, there was no sufficient evidence to conclude for surrogacy.

To the best of our knowledge, this is the second meta-analysis conducted in advanced STS patients, and the first on IPD. In the first meta-analysis, conducted on aggregated data, the authors reported a 0.61 trial-level association when assessing the surrogate properties of PFS, and concluded that PFS was an appropriate surrogate for OS [8]. However, we feel that data were insufficient to provide strong surrogacy evidence. No confidence interval for the trial-level association was reported, a key element to quantify the validity of a surrogate endpoint using appropriate criteria [24–26]. As the correlation estimate reported was derived from a smaller set of trials than in our study, it is likely that the precision was also poor.

Our study also presents some limitations. Some of the trials included date back to the 90 s. This should be regarded as a weakness, particularly with respect to the histological diagnosis. For instance, gastro-intestinal stromal tumors were considered as STS and included in some older trials. Additionally, the criteria for response assessment in solid tumors evolved in the past 20 years (WHO criteria, RECIST criteria). Finally, we could not include all relevant trials, as some of the sponsors and/or PIs did not agree to share their data. This is a recurrent challenge when performing a meta-analysis [34]. Trial design for advanced sarcoma is particularly challenging due to the rarity and the heterogeneity of the disease and treatments, which may contribute to weaken the observed correlations between candidate surrogates and OS [27]. Most STS trials include different clinical phenotypes to increase their statistical power, even though specific RCTs would be required [27–29]. In the present study, the distribution of sarcoma subtypes across trials was highly variable, with proportions ranging from 0% [20] to 18% [16] for liposarcoma, 18% [19] to 100% [20] for leiomyosarcoma and 0% [20] to 14% [22] for synovial sarcoma. Locally advanced and metastatic patients have different prognoses, yet they are often conflated in trials as “advanced” sarcomas. Heterogeneity in terms of sarcoma subtypes might benefit the treatment in terms of OS, but, pre-supposing the drug is inactive, not in terms of PFS. Additionally, assuming the treatment is mainly efficient on specific histological subtypes, the treatment effects estimations might be diluted when all subtypes are combined. Heterogeneity in terms of treatment settings remains between the trials included in our study, which could also have weakened the association between the candidate surrogates and OS. Indeed, 11 trials included only first-line treatment (79% of all patients), one trial included first and second line treatments (3% of patients), one trial included 2nd line treatment only (3% of patients) and one trial included second to fifth line treatments (13% of patients). Central review at study entry is also likely to interfere. Patients with inappropriate histological subtypes or grades could be included and thus dilute the associations. A majority of the trials included in our study performed radiological central review, and some reviewed histology locally or in a specialized center. Results from our sensitivity analysis on doxorubicin- or ifosfamide-based therapies as first-line metastatic treatment did not significantly differ from our main analysis. We estimated a higher trial-level association between the surrogate candidates and OS when focusing on patients with leiomyosarcomas. However, the individual-level associations were lower than in the primary analysis. This could be due to the limited number of patients included in the subgroup analysis. As a result, even though the results might seem promising, a larger IPD meta-analysis specific to leiomyosarcomas would be necessary to properly conclude. These factors should be accounted for when interpreting the statistically non-significant correlations observed in this meta-analysis.

Finally, absence of surrogacy could also be explained if the candidate endpoints (PFS, TTP, TTF) do not truly predict OS. Indeed, Booth and Eisenhauer, for example, have questioned the mechanisms of actions of some agents, especially those targeting cell signaling and angiogenesis, and whether with chronic administration, they could delay progression for a time but lead to evolutionary changes in tumors, producing a more aggressive phenotype after treatment, thus offsetting the earlier delay in progression [30].

In addition, absence of surrogacy evidence might be related to an absence of the treatment effect on OS. The Accelerated Approval regulations, instituted by the Food and Drug Administration (FDA) in 1992, allow drugs for serious conditions that filled an unmet medical need to be approved based on a surrogate endpoint. Using a surrogate endpoint enabled the FDA to approve these drugs faster. As a result, an increasing number of anticancer drug product approvals by the FDA are made based on endpoints other than OS [1, 31, 32], some with no sufficient proof of their surrogate validity for OS [32]. In advanced STS for instance, FDA granted approval for pazopanib in 2012 based on proof of benefit for PFS [18], even though at the time no study had assessed trial-level association between PFS and OS. In the context of accelerated approval, the FDA guidance on the term unmet medical need is imprecise. While this is not an issue in advanced sarcoma, recent data have shown that this term can often be overused [33], and as such the use of surrogates through pathways such as accelerated approvals, may be far greater than conditions with true unmet needs [34]. This issue is well illustrated with the example of bevacizumab in metastatic breast cancer; following accelerated approval based on PFS results, this drug was subsequently withdrawn following the publication of OS data [35, 36].

Several conditions have to be met to ensure adequate validation of a surrogate endpoint: (i) a significant quantity of high-quality data, both in terms of trials and patients, (ii) homogeneity, in terms of disease, settings, and mechanisms of action of the drugs, and (iii) strong statistical thresholds. Our meta-analysis did not lead to the validation of a surrogate in advanced sarcoma, as such OS should remain the primary endpoint in phase III trials evaluating systemic treatments in metastatic STS. Nevertheless, we highlighted moderate individual-level associations between the surrogate candidates and OS. These alternative endpoints thus remain useful in testing new treatments [37] in earlier drug development stages, such as phase II trials, or in futility assessment [38, 39].

## MATERIALS AND METHODS

This study is registered on the clinical trial registry clinicaltrials.gov (identifier: NCT02873923).

### Study selection

We identified trials by using a computerized search on MEDLINE with the following search algorithm: “sarcoma” [MeSH] AND “randomized controlled trial” [Text Word] AND trial [Text Word]. We limited our research to trials published before April the 7th, 2016. We also searched for trials on ClinicalTrials.gov and by contacting European sponsoring groups (EORTC, UNICANCER). Trials were eligible if they met the following criteria: (i) phase II or III randomized trials on humans, (ii) evaluating therapies for adults with advanced (i.e. locally advanced or metastatic) STS, (iii) at least one time-to-event endpoint other than OS as outcome, (iv) published or soon to be published in French or English, (v) signed agreement from the principal investigator and the sponsor, and (vi) available IPD.

### Patients, data and outcomes

We gathered IPD from all eligible trials. We assessed the surrogate properties of PFS, TTP and TTF evaluated at six and twelve months for 18-month OS. Outcomes were defined following the international DATECAN guidelines [40]. When none of the events included in the definition was observed, 6- and 12-month PFS, TTP and TTF were censored at the date of last follow-up or 6 months, respectively 12 months, of follow-up whichever came first.

### Surrogacy measures

The individual-level surrogacy was assessed following a copula-based approach [4]. The individual-level associations were estimated by the Spearman rank correlation coefficient (ρ_Spearman_) calculated from the copula parameter.

The trial-level surrogacy - the association between the treatment effects - was evaluated with two frameworks. Using a weighted linear regression model (WLR), treatment effects on OS and PFS/TTP/TTF were estimated separately for each trial, based on the logarithm of the hazard ratios (log[HR]) using Cox proportional hazard models. We assessed the association between the treatment effects using the coefficient of determination (R^2^_WLR_) of a linear regression model weighted by the trial size. The second method follows the two-stage model (2SM) adapted to time-to-event endpoints introduced by Burzykowski *et al.* [4]. We first simultaneously estimated the treatment effects on OS and on the candidate surrogate endpoints in each trial using a bivariate survival model based on a one-parameter Clayton copula. This approach enables taking into account the correlation between the endpoints in the estimation of the HR. We then estimated the association between the treatment effects (Weibull-distribution-based log[HR]) using an error-in-variable model, which allows taking into account the estimation errors. We assessed the trial-level association using the coefficient of determination (R^2^_2SM_).

All analyses were on an intention-to-treat basis. We reported confidence intervals for a 95% two-sided confidence-level (95% CI). All analyses were performed using SAS software v9.3 following Burzykowski *et al.* [4].

### Strength of association

The strength of the trial-level association was ranked according to the Institute for Quality and Efficiency in Health Care (IQWiG) guidelines [24]: high association (lower limit of the 95% CI of R^2^ ≥ 0.72), low association (higher limit of the 95% CI of R^2^ ≤ 0.49) or medium association (neither low nor high), meaning that the validity of the surrogate remains unclear.

### Subgroup analyses

To control for trials’ heterogeneity, we performed two subgroup analyses. Firstly, we retained only trials focusing on first line treatment that included doxorubicin- or ifosfamide-based therapies in the control arm. In the second analysis, we included only patients with leiomyosarcomas.

## CONCLUSIONS

Even though we highlighted moderate individual-level associations between the surrogate candidates and OS, our meta-analysis did not lead to significant evidence to validate PFS, TTP or TTF as surrogate markers for OS when assessing systemic treatment in advanced STS. PFS, TTP and TTF can be used as primary endpoints in phase II trials or as futility endpoints in phase III trials. However, OS should remain the primary endpoint in phase III trials until sufficient proof of surrogacy is provided. To achieve that goal, improvement in the conduct of sarcoma trials, particularly regarding the selection of histological subtypes, is necessary.

### Ethics approval and consent to participate

Not applicable.

### Availability of data and material

The datasets used and/or analyzed during the current study are available from the corresponding author on reasonable request.

## SUPPLEMENTARY MATERIALS TABLES


