# Supplementary material for: Surrogate endpoints in advanced sarcoma trials: a meta-analysis
Source: Oncotarget. 2018 Oct 2;9(77):34617–27. doi: 10.18632/oncotarget.26166 (PMC6195375; doi:10.18632/oncotarget.26166)
Supplement: Supplementary file 1 [file oncotarget-09-34617-s001.pdf]

Surrogate endpoints in advanced sarcoma trials: a meta-analysis

SUPPLEMENTARY MATERIALS

Supplementary Table 1: Number of events observed for progression-free survival, time-to-progression and time-to-treatment failure after 6 and 12 months of follow-up; All trials (Ntrial = 15; Npatient = 2846)*

Follow-up	Endpoint	Number of events
6 months	TTP	1629
	TTF	1646
	PFS	1693
12 months	TTP	2226
	TTF	2245
	PFS	2303

*PFS = progression-free survival; TTF = time-to-treatment failure; TTP = time-to-progression.

Supplementary Table 2: Number of events observed for progression-free survival, time-to-progression and time-to-treatment failure after 6 and 12 months of follow-up; Trials with doxorubicin- or ifosfamide-based control arm, first-line setting (Ntrial = 11; Npatient = 2243)

Folow-up	Endpoint	Number of events
6 months	TTP	1203
	TTF	1220
	PFS	1264
12 months	TTP	1695
	TTF	1714
	PFS	1768

*PFS = progression-free survival; TTF = time-to-treatment failure; TTP = time-to-progression.
